# Identification of candidate genes responsible for the susceptibility of apple (*Malus* × *domestica* Borkh.) to Alternaria blotch

**DOI:** 10.1186/s12870-019-1737-7

**Published:** 2019-04-08

**Authors:** Shigeki Moriya, Shingo Terakami, Kazuma Okada, Taku Shimizu, Yoshihiko Adachi, Yuichi Katayose, Hiroko Fujisawa, Jianzhon Wu, Hiroyuki Kanamori, Toshiya Yamamoto, Kazuyuki Abe

**Affiliations:** 1 Apple Research Station, Institute of Fruit Tree and Tea Science, National Agriculture and Food Research Organization (NARO), 92-24 Shimokuriyagawa Nabeyashiki, Morioka, Iwate 020-0123 Japan; 2 Institute of Fruit Tree and Tea Science, NARO, 2-1 Fujimoto, Tsukuba, Ibaraki 305-8604 Japan; 3 Citrus Research Station, Institute of Fruit Tree and Tea Science, NARO, 485-6 Okitsunaka-cho, Shimizu, Shizuoka City, Shizuoka 424-0284 Japan; 4 Advanced Genomics Breeding Section, Institute of Crop Science, NARO, 1-2 Ohwashi, Tsukuba, Ibaraki 305-8634 Japan

**Keywords:** *Alternaria alternata*, Bacterial artificial chromosome library, Disease resistance, Host-selective toxin, Guard model

## Abstract

**Background:**

The mechanism underlying the interaction between host plant and host-selective toxin (HST)-producing *Alternaria alternata* during infection is of particular interest for sustainable crop production. Alternaria blotch of apple (*Malus* × *domestica* Borkh.) caused by *A. alternata* apple pathotype is a major disease particularly in East Asia, which is the largest producer of apples globally. A single dominant gene, *Alt*, controls the susceptibility of the apple cultivar ‘Delicious’ to Alternaria blotch. In this study, we fine mapped the *Alt* locus and characterized three potential candidate genes.

**Results:**

We used 797 F_1_ individuals derived from 15 crosses between apple accessions susceptible (*Alt*/*alt*) and resistant (*alt*/*alt*) to Alternaria blotch to construct physical and genetic maps of the *Alt* locus located on the top of chromosome 11. Susceptible accessions were derived from ‘Delicious.’ To fine map the *Alt* locus, we constructed a BAC library of ‘Starking Delicious,’ a sport of ‘Delicious,’ and used graphical genotyping to delimit the *Alt* locus to a region of 43 kb. Three genes predicted within the candidate *Alt* region were potentially involved in plant defense response, among which the gene encoding a coiled coil-nucleotide binding-leucine rich repeat (CC-NB-LRR) type disease resistance protein was the most promising. Moreover, a 12-bp insertion was uniquely identified in the 5′ untranslated region of the *Alt*-associated allele of this gene, the presence or absence of which co-segregated with the susceptibility or resistance to *A. alternata* apple pathotype, respectively, among 43 tested cultivars including old ones and founders of modern apple breeding.

**Conclusion:**

A disease resistance protein has been suggested as a determinant of susceptibility/resistance to HST-producing *A. alternata* for the first time. Our finding provides new insight into the mechanism of HST-mediated disease control used by *A. alternata* against host plants.

**Electronic supplementary material:**

The online version of this article (10.1186/s12870-019-1737-7) contains supplementary material, which is available to authorized users.

## Background

Plant–pathogen interactions are of major interest for sustainable crop production with minimal use of chemicals. While most *Alternaria* species are saprophytic fungi that exist in soil or on decaying tissues [1, 2], host-selective toxin (HST) producing *Alternaria alternata* has acquired pathogenic traits and cause disease in a wide range of host plants. HST-producing *A. alternata* has seven pathogenic variants (pathotypes), each producing different HSTs specific for genera belonging to Rosaceae (*Malus*, *Pyrus*, *Fragaria*) and Rutaceae (*Citrus*) as well as Solanaceae species (tomato [*Solanum lycopersicum*] and tobacco [*Nicotiana tobacum*]) [3, 4]. HSTs are low molecular weight secondary metabolites that are toxic only to the host of the fungus producing the toxin but do not affect other plants.

While fungal pathogenicity has been extensively studied, the susceptibility of hosts to fungal infection remains poorly understood. Chemical structures have been elucidated for six of the seven HSTs, and the HST biosynthetic genes have been cloned, with the exception of the tobacco pathotype [3, 4]. Moreover, complementary experiments using *A. alternata* mutants that vary in their ability to produce HST have revealed the mechanism underlying its pathogenicity against host plants [5–7]. While the primary target sites of HSTs have been identified [3, 4], genes that determine host susceptibility have been identified only in rough lemon (*Citrus jambhiri* Lush.) and tomato [8, 9]. Therefore, elucidation of the mechanism underlying host susceptibility/resistance is needed to obtain a comprehensive understanding of the relationship between plant hosts and HST-producing *A. alternata*.

*A. alternata* apple pathotype (previously known as *A. mali* Roberts.) produces AM-toxin [10–12] and causes Alternaria blotch in apple (*Malus* × *domestica* Borkh.). AM-toxin has three related molecular species, AM-toxins I, II, and III, among which AM-toxin I is the most abundant. AM-toxin biosynthetic genes, *AMT1*, *AMT2*, *AMT3*, and *AMT4*, have been cloned and are clustered together in a small conditionally dispensable chromosome with many transposon-like sequences [6]. Alternaria blotch affects apple orchards in East Asia, which surpasses North America, and Europe as the region with the highest production of apples [13, 14]. It is characterized by the appearance of circular brown or blackish spots on leaves in late spring to early summer leading to defoliation. This results in reduction of photosynthesis and deterioration of fruit quality, leading to significant losses in apple production.

Although the level of susceptibility of apple cultivars to Alternaria blotch varies, the cutivars ‘Delicious,’ ‘Indo,’ and their sports and descendants are particularly susceptible [15]. Susceptibility to Alternaria blotch is controlled by the dominant gene *Alt*, which is mapped to chromosome 11 of ‘Starking Delicious’ (SD), a sport of ‘Delicious’ [16, 17]. Resistant cultivars are of the genotypes *alt*/*alt*, and susceptible cultivars are either *Alt*/*alt* or *Alt*/*Alt* although cultivars homozygous for *Alt* have not been identified [16]. Several simple sequence repeat (SSR) markers have been previously identified as flanking the *Alt* locus, which serve as good starting points for the positional cloning of *Alt*. In this study, we performed fine mapping of *Alt* using F_1_ progenies derived from crosses between apple accessions resistant and susceptible to Alternaria blotch. All susceptible accessions used in this study are derivatives of ‘Delicious.’ A bacterial artificial chromosome (BAC) library of ‘SD’ was constructed for fine mapping the *Alt* locus. Furthermore, a PCR-based genotyping marker was designed for scoring the resistance/susceptible phenotype of apple cultivars.

## Results

### Fine mapping of *Alt*

For fine mapping the *Alt* locus with 797 F_1_ plants (Table 1, Additional file 1: Figure S1), we first applied a marker enrichment approach for the genetic map of chr 11 constructed from two reciprocal full-sibs of ‘SD’ and ‘Jonathan.’ Forty-five SSR markers described previously [17] were tested, of which 25 markers were polymorphic in ‘SD’ and were subjected to linkage analysis (Additional file 2: Table S1).

**Table 1:** F_1_ progenies used for fine mapping of *Alt*

Maternal parent^a^	Paternal parent	Abbreviation	No. of individuals	No. of recombinants^b^
Starking Delicious	Jonathan	SDJ	57	0
Jonathan	Starking Delicious	JSD	57	3
Sansa	Starking Delicious	P1	39	2
Starking Delicious	Sansa	P2	46	2
Sansa	Redgold	P3	36	2
Jonathan	Redgold	P4	40	3
Redgold	Jonathan	P5	27	0
Gala	Morioka 61	P6	50	2
Sansa	Morioka 61	P7	57	2
Morioka 61	5–6393	P8	3	0
4–547	Morioka 61	P9	26	0
4–161	5–5102	P10	81	1
Sensyu	Morioka 61	P11	86	1
Morioka 59	5–3645	P12	38	0
Morioka 61	Silken	P13	154	5
	Total		797	23 (2.9%)

^a^Apples shown with an underline are susceptible (*Alt*/*alt*) to Alternaria blotch. Morioka 59: Kitakami × Hatsuaki, Morioka61: Tsugaru × Kitakami, 5–6393: Akane × 4–23 (Fuji × [Golden Delicious × Indo]), 4–547: Fuji × Hatsuaki, 4–161: Hatsuaki × Starking Delicious, 5–3645: Sansa × Tsugaru, 5–5102: Tsugaru × Fuji. A graphical illustration of the pedigree of susceptible apples is also shown in Additional file 1: Figure S1

^b^Individuals in which recombination occurred between Mdo.chr11.27 and Mdo.chr11.44

**Fig. 1:**
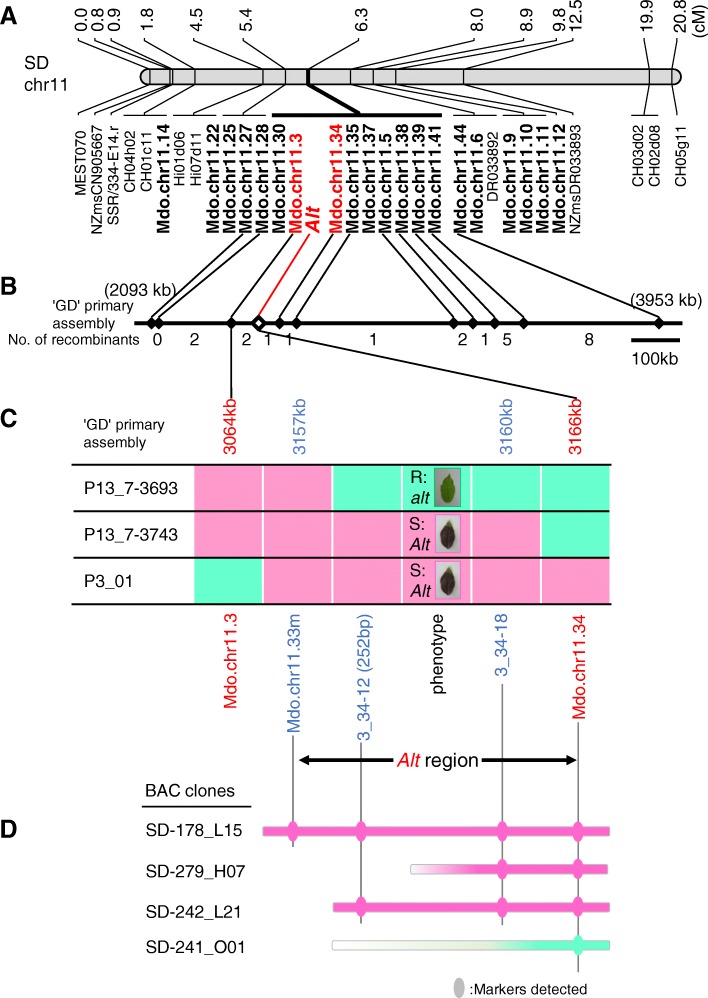
Fine mapping of the *Alt* locus. **a** Marker-enriched initial map of ‘Starking Delicious’ (SD) constructed from 114 individuals of reciprocal crosses between ‘SD’ and ‘Jonathan.’ Simple sequence repeat (SSR) markers developed in this study are indicated in bold. Chr: chromosome. **b** Detailed physical map of *Alt* with genetic information using 797 F_1_ individuals derived from 15 crosses. The physical position of each marker is based on the ‘Golden Delicious’ genome version 1.0 primary assembly. Graphical representation of the genotype is also shown in Additional file 3: Table S2. **c** Final graphical genotype of the sequence flanking the *Alt* locus. Only three key recombinants are shown. The candidate region of *Alt* is indicated using double-headed arrow. Green and pink segments indicate *Alt*- and *alt*-associated regions, respectively. **d** Screening of bacterial artificial chromosome (BAC) clones of ‘SD’

Of the 25 SSR markers, 21 mapped to chr 11. Of these 21 SSRs, 9 co-segregated with the *Alt* locus (Fig. 1a). The SSR markers, Mdo.chr11.28 and Mdo.chr11.44 flanked the *Alt* locus on either side. However, because Mdo.chr11.28 was less polymorphic, Mdo.chr11.27 was used together with Mdo.chr11.44 for further fine mapping of the *Alt* locus. Among 683 F_1_ individuals, 20 plants showing recombination between Mdo.chr11.27 and Mdo.chr11.44 were identified (Table 1 and Additional file 3: Table S2). Of these 20 recombinants plus an additional three recombinants derived from ‘SD’ and ‘Jonathan’ reciprocal full-sibs, 9 and 14 plants were identified as susceptible and resistant to Alternaria blotch, respectively (Table 1). None of the plants exhibited moderate resistance. Exploiting the phenotypic and the genetic linkage data, the *Alt* region was represented as a graphical genotype (Fig. 1b and Additional file 3: Table S2). The candidate region of *Alt* was identified between Mdo.chr11.3 and Mdo.chr11.34, spanning a physical distance of 102 kb according to the ‘Golden Delicious’ genome version 1.0 primary assembly. Three plants showing recombination between *Alt* and either Mdo.chr11.3 or Mdo.chr11.34 were identified.

To narrow down the candidate region further, we designed a new SSR marker Mdo.chr11.33 m by redesigning the primer sequence of Mdo.chr11.33 which was designed from the region between Mdo.chr11.3 and Mdo.chr11.34 and did not amplify in the marker enrichment approach and even developed two additional SSR markers (3_34–12 and 3_34–18) between Mdo.chr11.3 and Mdo.chr11.34 (Table 2). To identify *Alt*-associated alleles, we initially mapped these markers to the ‘SD’ map (data not shown). For markers Mdo.chr11.33 m and 3_34–18, 267-bp and 299-bp fragments were PCR amplified, respectively, both of which represented the *Alt*-associated alleles. For the marker 3_34–12, two fragments (288 and 252 bp) representing the *Alt*-associated alleles were PCR amplified, suggesting that primer pairs of 3_34–12 amplified two tightly linked loci (3_34–12 [288 bp] and 3_34–12 [252 bp]). We tested these SSR markers on 23 recombinants and identified that 3_34–12 (252 bp) and 3_34–18 co-segregated with *Alt* (Fig. 1c). Mdo.chr11.33 m was located between *Alt* and Mdo.chr11.3. The 3_34–12 (288 bp) locus mapped to a genomic location between Mdo.chr11.3 and Mdo.chr11.28, outside the candidate *Alt* region (Additional file 3: Table S2). Overall, fine mapping delimited *Alt* to a 9-kb region between Mdo.chr11.33 m and Mdo.chr11.34 according to the ‘Golden Delicious’ genome version 1.0 primary assembly.

**Table 2:** Novel SSR markers developed in this study from apple contigs

Marker	Primer sequence (5′ → 3′)	Origin of apple contig	Contig start position (bp)	SSR start position in contig (bp)	Motif type	Copy number
Mdo.chr11.33 m	F: GTTCGATCGGGGTGAAAGT R: CCCCATCCATTTACCCTACC	MDC021160.220	3,150,224	6672	GA	15.5
3_34–12	F: CCAATTGAAGACCTCCCAAA R: CCAGGAAAAGGACGCTACTG	MDC004702.449	3,152,413	4269	TAA	5.7
3_34–18	F: GAATCCCGAACTGAACCAAA R: GCTAAAATTTGGGCTTTAGGC	MDC001844.206	3,160,267	223	AT	13

### Identification of BAC clones spanning the *Alt* region

**Fig. 2:**
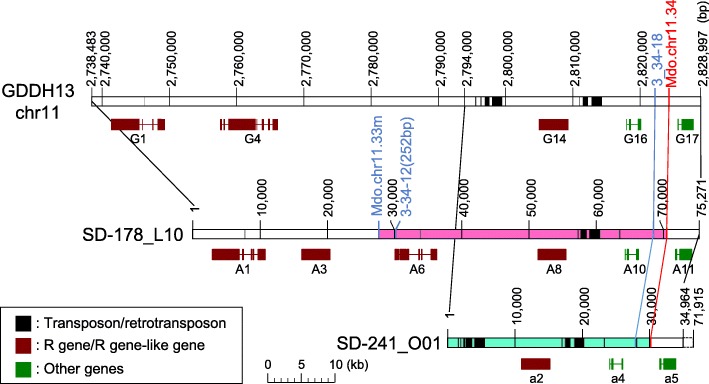
Genetic characterization of the *Alt* region. Partial chromosome 11 of GDDH13, and BAC clones SD-178_L15 (*Alt* associated) and SD-241_O01 (*alt* associated) are shown. Genes predicted using FGNESH are indicated

To identify genes in the candidate region of *Alt*, we constructed a BAC library of ‘SD’ with an average insert size of 180 kb and a total of 49,920 BAC clones at the first trial. Thus, the size of the BAC library is approximately 8985 Mb, which is approximately 11 times the size of the apple genome. However, clones harboring each of the four SSR markers (Mdo.chr11.3, Mdo.chr11.34, 3_34–12, and Mdo.chr11.33 m) could not be identified. Therefore, we performed the second trial with shorter fragments than the first trial and then developed an additional 61,056 BAC clones although their average insert size was not verified. Eventually, four BAC clones (SD-178_L15, SD-279_H07, SD-242_L21, and SD-241_O01) harboring at least one SSR marker were identified (Fig. 1d). The BAC clones, SD-178_L15, SD-279_H07, SD-242_L21 were identified as *Alt*-associated clones, whereas SD-241_O01 was identified as the *alt*-associated clone. Only one *Alt*-associated clone covered the complete candidate region (SD-178_L15), whereas the span of the *alt*-associated clone (SD-241_O01) was unconfirmed. Both SD-178_L15 and SD-241_O01 were shotgun sequenced and determined to span a length of 75,271 and 71,915 bp, respectively (Fig. 2). The *Alt* candidate region spanned approximately 43 kb, which was located within the range of 27–70 kb from the 5′-end of SD-178_L15. The BAC clone SD-241_O01 harbored Mdo.chr11.34 and only the forward primer of 3_34–18 at 30,344 bp and 28,015 bp from the 5′-end, respectively, but did not harbor the other two markers, 3_34–12 and Mdo.chr11.33 m.

### In silico gene identification and characterization

Sequences of the BAC clones SD-178_L15 and SD-241_O01 and the partial sequence of GDDH13, a new genome sequence derived from a ‘Golden Delicious’ double haploid line [18], were examined for the presence of genes, transposable elements, and other features. The structure and location of transposable elements were clearly distinct between SD-178_L15, SD-241_O01, and GDDH13 (Fig. 2). We identified 11 (A1–11), 12 (a1–12), and 17 (G1–17) predicted ORFs in SD-178_L15, SD-241_O01, and GDDH13 (Table 3 and Fig. 2). Protein sequences of several predicted ORFs showed high similarity to those of genes involved in plant defense responses (Table 3). InterProScan identified a leucine-rich repeat (LRR) domain, a major component of disease resistance (R) genes, in four, three, and three ORFs of SD-178_L15, SD-241_O01, and GDDH13, respectively (Table 4 and Fig. 2). RIN4, a pathogenic type III effector avirulence factor Avr cleavage site, was also identified in A10, a4, and G16 ORFs. The *Alt* candidate region of SD-178_L15 included five predicted ORFs (A6–10), of which three showed characteristic features of R genes and two represented retrotransposon-like sequences and were discarded from the analysis.

**Table 3:** ORFs predicted in this study from BAC clones and partial genomic sequence

BAC clone/genome	ORF	Transcription start (bp)	Transcription end (bp)	Deduced amino acid length (aa)	Strand	Homologous protein (Species)	*E*-value	Accession no.
SD-178_L15	A1	2061	11,998	1679	+	PREDICTED: putative disease resistance RPP13-like protein 1 (*Malus domestica*)	0.0	XP_008384572.2
	A2	15,932	13,969	203	–	hypothetical protein DOTSEDRAFT_19432 (*Dothistroma septosporum* NZE10)	0.24	EME48939.1
	A3	15,987	20,894	1411	+	PREDICTED: putative disease resistance RPP13-like protein 1 (*Pyrus* × *bretschneideri*)	0.0	XP_018507989.1
	A4	25,096	26,511	214	+	PREDICTED: uncharacterized protein LOC103409892 (*Malus domestica*)	5.00E-132	XP_008346905.1
	A5	27,488	28,157	40	+	None		
	A6	29,534	36,522	835	+	PREDICTED: putative disease resistance protein RGA3 isoform X2 (*Pyrus* × *bretschneideri*)	0.0	XP_009379454.1
	A7	43,448	45,613	168	+	Hypothetical protein COA78_37210 (*Blastopirellula* sp.)	0.47	PHR87371.1
	A8	50,640	55,659	1405	+	PREDICTED: putative disease resistance RPP13-like protein 1 (*Malus domestica*)	0.0	XP_008366183.1
	A9	55,769	57,610	296	+	Uncharacterized protein LOC110755101 (*Prunus avium*)	1.00E-104	XP_021811957.1
	A10	63,542	66,456	174	+	PREDICTED: uncharacterized protein LOC103967858 isoform X1 (*Pyrus* × *bretschneideri*)	2.00E-37	XP_009379452.1
	A11	71,752	74,786	650	+	PREDICTED: WEB family protein At5g55860 (*Malus domestica*)	0.0	XP_008384579.2
SD-241_O01	a1	Not identified	3754	222	+	PREDICTED: uncharacterized protein LOC108170510 (*Malus domestica*)	7.E-63	XP_017181004.1
	a2	10,358	15,380	1405	+	PREDICTED: putative disease resistance RPP13-like protein 1 (*Malus domestica*)	0.0	XP_008366183.1
	a3	15,499	17,345	322	+	Uncharacterized protein LOC110755101 (*Prunus avium*)	5E-132	XP_021811957.1
	a4	23,336	26,622	115	+	PREDICTED: uncharacterized protein LOC103967858 isoform X1 (*Pyrus* × *bretschneideri*)	1.00E-36	XP_009379452.1
	a5	31,018	34,478	650	+	PREDICTED: WEB family protein At5g55860 (*Malus domestica*)	0.0	XP_008384579.2
	a6	34,684	38,521	473	+	PREDICTED: uncharacterized protein LOC103447176 (*Malus domestica*)	0.0	XP_008384578.1
	a7	44,893	39,592	324	+	PREDICTED: phosphatidylinositol/phosphatidylcholine transfer protein SFH1-like (*Pyrus* × *bretschneideri*)	0.0	XP_009367226.1
	a8	51,499	52,557	69	–	PREDICTED: kinesin-like protein KIF1C (*Lates calcarifer*)	0.11	XP_018537717.1
	a9	59,979	54,964	609	+	PREDICTED: serine/threonine-protein kinase D6PK-like (*Pyrus* × *bretschneideri*)	0.0	XP_009367228.1
	a10	60,552	63,449	152	–	Pre-rRNA-processing protein TSR2-like (*Prunus avium*)	3.00E-66	XP_021818064.1
	a11	69,641	64,724	353	–	PREDICTED: DNA-directed RNA polymerases I and III subunit rpac1-like (*Malus domestica*)	3.00E-164	XP_008349490.1
	a12	69,866	Not Identified	325	–	PREDICTED: putative disease resistance RPP13-like protein 1 (*Malus domestica*)	0.0	XP_008384580.1
GDDH13 Chr 11 partial	G1	2,740,584	2,749,771	1689	+	PREDICTED: putative disease resistance RPP13-like protein 1 (*Malus domestica*)	0.0	XP_008384572.2
	G2	2,754,217	2,752,730	125	–	Putative reverse transcriptase family member (*Malus domestica*)	2.00E-36	CBL94165.1
	G3	2,754,290	2,757,403	577	–	Putative COBL7 (COBRA-LIKE 7) (*Malus domestica*)	0.0	CBL94184.1
	G4	2,757,523	2,766,112	1770	+	PREDICTED: putative disease resistance protein RGA3 isoform X2 (*Pyrus* × *bretschneideri*)	0.0	XP_009379454.1
	G5	2,769,661	2,774,105	1049	+	T4.15 (*Malus* × *robusta*)	0.0	CCH50976.1
	G6	2,774,818	2,774,245	133	–	T4.14 (*Malus* × *robusta*)	3.00E-89	CCH50975.1
	G7	2,774,858	2,778,564	786	+	Hypothetical protein (*Malus domestica*)	3.00E-149	AEJ72571.1
	G8	2,778,594	2,779,821	161	+	PREDICTED: uncharacterized protein LOC103420450 (*Malus domestica*)	8.00E-11	XP_008356739.1
	G9	2,781,742	2,780,939	49	–	None		
	G10	2,781,835	2,787,073	592	+	Putative COBL7 (COBRA-LIKE 7) (*Malus domestica*)	1.00E-145	CBL94184.1
	G11	2,787,274	2,788,139	225	+	T4.14 (*Malus* × *robusta*)	2.00E-144	CCH50975.1
	G12	2,791,526	2,788,282	1416	–	T4.15 (*Malus* × *robusta*)	0.0	CCH50976.1
	G13	2,794,998	2,799,499	430	+	PREDICTED: uncharacterized protein LOC103438444 (*Malus domestica*)	3.00E-135	XP_017188663.1
	G14	2,804,362	2,809,384	1405	+	PREDICTED: putative disease resistance RPP13-like protein 1 (*Malus domestica*)	0.0	XP_008366183.1
	G15	2,809,494	2,812,371	338	+	Uncharacterized protein LOC110755101 (*Prunus avium*)	6.00E-100	XP_021811957.1
	G16	2,817,267	2,820,181	174	+	PREDICTED: uncharacterized protein LOC103967858 isoform X1 (*Pyrus* × *bretschneideri*)	2.00E-37	XP_009379452.1
	G17	2,825,063	2,828,512	650	+	PREDICTED: WEB family protein At5g55860 (*Malus domestica*)	0.0	XP_008384579.2

**Table 4:** Functional domains identified in predicted ORFs in the *Alt* region

BAC clone/genome	Gene^a^	Functional domains (InterPro)	InterPro ID	Match position (aa)
SD-178_L15	A5	NP^a^		
	A6	RX-CC_like	CD14798	10..98
		Leucine-rich repeat domain superfamily	IPR32675	106..563
		F-box-like domain superfamily	IPR001810	641..691
	A7	NP		
	A8	RX-CC_like	CD14798	33..136
		P-loop containing nucleoside triphosphate hydrolase	IPR027417	160..425
		NB-ARC	IPR002182	173..451
		Winged helix-turn-helix DNA-binding domain superfamily	IPR011991	405..487
		Leucine-rich repeat domain superfamily	IPR32675	487..1393
	A9	Gag-polypeptide of LTR copia-type	IPR029472	16..59
	A10	RIN4, pathogenic type III effector avirulence factor Avr cleavage site	IPR008700	12..47
SD-241_O01	a1	Gag-polypeptide of LTR copia-type	IPR029472	30..70
	a2	RX-CC_like	CD14798	33..136
		P-loop containing nucleoside triphosphate hydrolase	IPR027417	161..425
		NB-ARC	IPR002182	173..451
		Winged helix-turn-helix DNA-binding domain superfamily	IPR011991	405..490
		Leucine-rich repeat domain superfamily	IPR032675	487..1393
	a3	Gag-polypeptide of LTR copia-type	IPR029472	16..59
	a4	RIN4, pathogenic type III effector avirulence factor Avr cleavage site	IPR008700	12..47
GDDH13	G4	RX-CC_like	CD14798	123..249
		P-loop containing nucleoside triphosphate hydrolase	IPR027417	275..544
		NB-ARC	IPR002182	304..570
		Winged helix-turn-helix DNA-binding domain superfamily	IPR011991	524..608
		Leucine-rich repeat domain superfamily	IPR032675	985..1428
		Receptor L-domain superfamily	IPR036941	1310..1427
		RIN4, pathogenic type III effector avirulence factor Avr cleavage site	IPR008700	1439..1474
		F-box-like domain superfamily	IPR036047	1532..1586
		F-box domain	IPR001810	1530..1570
	G5	Endonuclease/exonuclease/phosphatase superfamily	IPR036691	110..358
		SWR1-complex protein 5/craniofacial development protein	IPR027124	112..406
		Reverse transcriptase domain	IPR000477	621..867
	G6	NP		
	G7	NP		
	G8	NP		
	G9	NP		
	G10	NP		
	G11	NP		
	G12	Endonuclease/exonuclease/phosphatase superfamily	IPR036691	94..300
		SWR1-complex protein 5/craniofacial development protein	IPR027124	57..78
		SWR1-complex protein 5/craniofacial development protein	IPR027124	97..298
	G13	Gag-polypeptide of LTR copia-type	IPR029472	30..70
		GAG-pre-integrase domain	IPR025724	268..328
	G14	RX-CC_like	CD14798	33..136
		P-loop containing nucleoside triphosphate hydrolase	IPR027417	161..425
		NB-ARC	IPR002182	173..451
		Winged helix-turn-helix DNA-binding domain superfamily	IPR011991	405..487
		Leucine-rich repeat domain superfamily	IPR032675	487..1393
	G15	Gag-polypeptide of LTR copia-type	IPR029472	16..59
	G16	RIN4, pathogenic type III effector avirulence factor Avr cleavage site	IPR008700	12..47

^a^*NP* none predicted

**Fig. 3:**
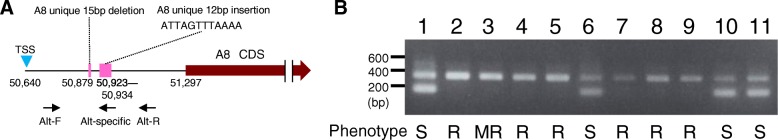
DNA marker for the detection of the *Alt*-associated allele of A8 ORF. **a** Schematic representation of the A8 ORF unique 12-bp insertion structure and primers for its detection. **b** Gel image showing PCR products amplified from the genomic DNA of apple cultivars with three primers “Alt-F,” “Alt-R,” and “Alt-specific.” Different lanes of the gel represent different apple cultivars: 1, ‘Starking Delicious’; 2, ‘Jonathan’; 3, ‘Golden Delicious’; 4, ‘Ralls Janet’; 5, ‘Worcester Pearmain’; 6, ‘Indo’; 7, ‘Cox’s Orange Pippin’; 8, ‘McIntosh’; 9, P13_7–3693; 10, P13_7–3743; and 11, P3_01. S = susceptible, R = resistant, MR = moderately resistant. TSS: transcription start site, CDS: coding sequences

Within the *Alt* candidate region, the A6 ORF was uniquely present on SD-178_L15, and was absent from SD-241_O01, and GDDH13. The A6 ORF showed high similarity to coiled coil-nucleotide binding-LRR (CC-NB-LRR) type proteins; however, it lacked the NB domain (Table 4 and Additional file 4: Figure S2). It showed the highest similarity (62%) to the predicted ORF MD11G1030400 located on 2.656 Mb of chr 11 of the GDDH13 genome. The A8, a2, and G14 ORFs showed high similarity to CC-NB-LRR class R proteins, clearly indicating that they belong to this category of protein. They represented highly conserved 1405 amino acids (> 99% similarity) encoding CC, NB, and LRR domains (Table 4) and were predicted by InterPro to be extracellular proteins (data not shown). The A8 ORF harbored several unique sequence variations, including several amino acid substitutions in the CC and NB domains (Additional file 5: Figure S3), and a 12-bp insertion and a 15-bp deletion in the 5′ untranslated region (UTR) (Fig. 3). The A10, a4, and G16 ORFs harbored RIN4, a pathogenic type III effector avirulence factor Avr cleavage site domain (Table 4). The amino acid sequences of A10 and G16 were identical, and their promoter sequences 2 kb upstream of the transcription start site were also identical, except for 1-bp mismatch. However, the predicted amino acid sequence of a4, especially at the C-terminus, differed significantly from that of A10 and G16 (Additional file 6: Figure S4).

### DNA marker for *Alt*

The unique insertion in the 5′ UTR of the A8 ORF was used to develop a PCR-based genotyping marker for the *Alt* region (Fig. 3). Accessions carrying the insertion were susceptible (score > 2.5) to infection with the AKI-3 isolate of *A. alternata* apple pathotype (Additional file 7: Table S3 and Fig. 3).

## Discussion

In the present study, we fine mapped the *Alt* locus to a 43 kb region on the top of chr 11 using BAC clones (Figs. 1 and 2) and predicted three ORFs as potential candidates underlying the susceptibility or resistance response to the *A. alternata* apple pathotype. One of these predicted ORFs encodes a typical R protein.

### Genes controlling Alternaria blotch susceptibility

An SSR marker (CH05g07) linked to a dominant gene conferring susceptibility to the *A. alternata* apple pathotype at a distance of 5.6 cM has been previously reported in ‘Golden Delicious’ [19]. However, because CH05g07 is located on two different chr (12 and 14) [20], the genomic position of the gene identified by Li et al. [19] possibly differs from that of the *Alt* locus mapped in the present study (Fig. 1). This inconsistency may be due to differences in genes responsible for the reaction to pathogen isolate attack or to the experimental conditions used. It may be that the virulence of *A. alternata* in the same cultivar differs between China and Japan [15, 21]. For example, ‘Golden Delicious’ has been defined as susceptible to Alternaria blotch in Chinese studies [19] but as resistant [15, 22, 23] or moderately resistant [24] in Japanese studies. The finding that application of HST at high concentrations to resistant cultivars induces susceptibility [25] suggests that pathogens used in those studies differ in their ability to produce HST; the Chinese one might produce AM-toxin in great quantities than the Japanese one. Therefore, it is important to characterize the virulence of the pathogens studied. Moreover, the long incubation time (7 days) used for the phenotypic evaluation of genotypes by Li et al. [19] compared with that used by Abe et al. [15] may raise other resistance/susceptibility systems controlled by genes that are not identical to the one identified in the present study. The mechanisms underlying these inconsistencies need to be investigated in future studies.

### Candidate gene identification

No *alt*-associated BAC clone was found to completely span the *Alt* region, suggesting that the nature of the sequence or chromosomal structure of this region inhibited its cloning. One possibility could be that the region surrounding the *Alt* candidate region contains many *Hin*dIII recognition sites, and this impeded the formation of long fractions we harvested at the first trial. The in silico ORF prediction revealed several R genes in BAC clones and the GDDH13 partial genome sequence. This result is consistent with previous observations of the CC-NB-LRR gene cluster at the top of chr 11 [26]. Although the predicted ORFs were not verified using RT-PCR, we predicted two ORFs encoding R proteins (A6 and A8) and one encoding a defense response-related protein (A10) within the candidate region (Table 3 and Fig. 2). Amino acid sequences of both A6 and A8 showed high similarity to CC-NB-LRR class R proteins; however, because A6 lacked the NB-ARC domain that is typical and critical for R protein function in this class of genes [27], A6 is probably a pseudogene. The absence of A6 allele in SD-241_O01 and GDDH13 could be explained by duplication of NB-LRR gene. In the vicinity of A6, the non-coding nucleotide sequence of SD-178_L10 is very different from those of GDDH13 and SD-241_O01 (data not shown); there is almost no similarity between SD-178_L10 and the other two, suggesting ectopic recombination and/or gene translocation, which are the driving forces behind enlargement of the resistance gene analogous (RGA) family, including NB-LRR genes, in the plant genome. The fact that A6 showed the highest similarity to the MD11G1030400 located approximately 100 kb from A6 is in line with the findings of a previous study [28]; NB-LRR pseudogenes are often present within 100 kb of the bonafide NB-LRR gene. Therefore, it could be possible that A6 was generated by gene duplication and is in the process of defunctionalizing.

Because the predicted ORFs A8, a2, and G14 were considered to be CC-NB-LRR class R genes, the encoded proteins could recognize an effector directly or indirectly, thus inducing a hypersensitive response (HR). It was suggested that several amino acid substitutions in the CC and NB domains could be associated with changes in their functions. The high conservation of amino acid sequences of these ORFs (> 99% similarity) suggests A8 to be a functional R gene, and unique polymorphisms in its 5′ UTR imply the distinct expression profile of the *Alt*-associated allele, although the future research is necessary to collect evidence supporting this theory.

The ORFs, A10, a4, and G16 were predicted to encode RIN4, a pathogenic type III effector avirulence factor Avr cleavage site (Table 4). In *Arabidopsis thaliana*, *RIN4* mediates HR against *Pseudomonas syringae* and is associated with the well-studied plant–pathogen relationship called the “guard model” [29–31]. The R proteins, RPM1 and RPS2 (i.e., guards), monitor RIN4 (i.e., guardee) invariability. The attack by pathogenic effectors secreted by *P. syringae* modifies RIN4, thereby activating RPM1 and RPS2 and inducing HR. A similar mechanism could exist in apple, where A10, a candidate of *Alt*, would function as a guardee protein*.* However, because sequences of A10 and G16 predicted proteins and promoters were highly conserved, it suggests that there are no functional and expression level differences between them and therefore do not appear to be plausible candidates for the *Alt* locus.

Consequently, among the three predicted in the SD-178_L15 BAC clone, A8 appears to be the most promising candidate for the *Alt* locus. These findings also suggest the elicitor activity of AM-toxin. Although the suppressor effect of AM-toxin is well documented [32], the elicitor activity of AM-toxin has been described for the first time in this study.

### Predicted mechanism underlying susceptibility control

Despite counter evidence, HR is thought as a plants’ defense mechanism against invading pathogens whereby the pathogen is contained within the dead tissue by inducing programmed cell death in areas surrounding the site of infection [33]. In typical relationships between plants and fungal pathogens mediated by R genes, resistance is usually dominantly inherited as a consequence of gene-to-gene interaction. By contrast, the relationship between apple and *A. alternata* apple pathotype exhibits an opposite trend, whereby Alternaria blotch susceptibility is dominantly inherited [16]. This is similar to the relationship between *A. thaliana* and *Cochliobolus victoriae* [34]. *C. victoriae* is a necrotrophic fungus that produces the HST victorin that affects *A. thaliana*. The susceptibility of *A. thaliana* to *C. victoriae* is dominantly inherited [35], which can be explained on the basis of the guard model [36, 37]. Lorang et al. [38] identified the susceptibility gene, *LOV1* that encodes a CC-NB-LRR class R protein. LOV1 guards TRXh5, a target of victorin. The attack by victorin on TRXh5 increases free LOV1, resulting in LOV1-mediated HR. Thus, *C. victoriae* exploits the plants’ defense response for its own pathogenesis [34]. Because the present study also suggested the involvement of R genes in the infection by HST-producing *A. alternata*, it is possible that the relationship between apple and HST-producing *A. alternata* is similar to that observed between *A. thaliana* and *C. victoriae*.

Tabira et al. [39] indicated that cell death is not necessary for the infection of apple by *A. alternata* apple pathotype, suggesting that a certain step of HR induction is sufficient for *A. alternata* infection. It is noteworthy that *A. alternata*, a saprophyte, mimics biotrophic pathogens and elicits a resistance response to exploit the hosts’ defense system for its invasion. However, details of the molecular mechanisms underlying the infection of apple by *A. alternata* apple pathotype need to be elucidated.

### Comprehensive understanding of the relationship between *A. alternata* and its host

The relationship between HST-producing *A. alternata* and host plants among the Rosaceae is similar. For example, black spot disease of Japanese pear and that of strawberry (*F*. × *ananassa* Duch.) is caused by the Japanese pear and strawberry pathotypes of *A. alternata*, respectively [3, 4]. It has been shown that the susceptibility of host plants to the disease is dominantly inherited, similar to the Alternaria blotch in apple [16, 40, 41]. HSTs produced by these pathotypes target the plasma membrane of the host cells [4]. In Japanese pear, the susceptibility gene *A* has been mapped to pear chr 11 between the markers Mdo.chr11.28 and Mdo.chr11.34 [17], thus perfectly corresponding to the *Alt* candidate region in apple; the chromosomal location of the susceptibility gene in strawberry is unknown. The *A. alternata* apple pathotype has recently been shown to cause black spot disease in the European pear (*P. communis* L.) [42]. Although the inheritance pattern has not been studied, AM-toxin induces veinal necrosis in leaves of specific European pear cultivars, such as ‘Le Lectier’ and ‘General Leclerc’ but not in ‘Bartlett’ [42], suggesting the existence of a similar HST-producing *A. alternata* – host relationship in the European pear. Based on these findings, we hypothesize that *A. alternata* exploits plant defense systems to express pathogenicity against the Rosaceae family, and that genes of Roseaceae hosts involved in susceptibility to *A. alternata* may be functionally conserved.

### Marker-assisted breeding of Alternaria blotch-resistant apple

Using three primers (Alt-F, Alt-R, and Alt-specific), we were able to score the *Alt* genotype of modern breeding founders and old cultivars, indicating the utility of this marker for the breeding of Alternaria blotch-resistant apple. Co-segregation of the 12-bp insertion with susceptible phenotype suggests single origin of susceptibility to Alternaria blotch and sufficient linkage disequilibrium between the insertion and causal polymorphism(s) of *Alt*. However, it did not discriminate moderately resistant cultivars such as ‘Golden Delicious’ from resistant cultivars, which needs to be investigated in future research.

## Conclusion

A CC-NB-LRR class R protein was identified as a promising candidate gene for *Alt*. This is the first study that identified a candidate gene involved in HR induction for the control of susceptibility/resistance to HST-producing *A. alternata* diseases. This finding provides new insights into the relationship between HST-producing *A. alternata* and host plants. This information will be useful in the development of better disease control strategies and will also improve our understanding of the co-evolution of plant defense mechanisms and fungal pathogenicity. Most importantly, the DNA marker developed in this study serves as a tool for marker-assisted breeding of Alternaria blotch-resistant apple.

## Methods

### Plant materials and DNA extraction

A total of 797 F_1_ plants derived from 15 crosses between Alternaria blotch-susceptible (*Alt*/*alt*) and -resistant (*alt*/*alt*) apple accessions were used in this study (Table 1). This included 114 F_1_ plants derived from reciprocal crosses between ‘SD’ and ‘Jonathan’ that have been previously assessed for their resistance to Alternaria blotch [43]. The susceptible accessions used in this study were derived from the common founder, ‘Delicious’ (susceptible, *Alt*/*alt*; Additional file 1: Figure S1). Moreover, eight founders of modern world apple cultivars and 35 old world apple cultivars described previously [15] were also included in this study (Additional file 7: Table S3). All apple genotypes were grown and maintained at the Apple Research Station, Institute of Fruit Tree and Tea Science, NARO, Morioka, Japan.

Genomic DNA was isolated from of all F_1_ progenies and their parents. Briefly, 100 mg of young leaves were ground in liquid nitrogen and incubated with 1 ml of an isolation buffer (10% PEG 6000, 100 mM Tris-HCl [pH 8.0], 350 mM sorbitol, and 50 mM EDTA [pH 8.0]) at 50 °C for 30 min. Genomic DNA was extracted using the DNA extraction device, PI-50α (Kurabo, Osaka, Japan) in accordance with the manufacturer’s instructions.

### Evaluation of Alternaria blotch resistance

The monoconidial isolate of *A. alternata* apple pathotype, AKI-3 was used for the evaluation of Alternaria blotch resistance. Inoculation was performed using the detached-leaf method as described previously [15]. Briefly, five, second or third youngest leaves, were removed from the growing shoots of each plant and coated with a suspension of 2 × 10^5^ conidia of the AKI-3 isolate using a mist sprayer. The inoculated leaves were incubated at 20 °C for 48 h in the dark in a plastic chamber. The resistance level of each leaf was scored on a scale of 0 (no visible symptoms) to 5 (almost complete necrosis of the whole leaf) as described previously [15]. To determine the resistance level of a genotype, resistance scores of all leaves belonging to the same genotype were averaged. Scoring of the resistance level of genotypes was slightly modified from Abe et al. [15]; a genotype was categorized as resistant: mean score ≤ 0.5, moderately resistant: mean score 0.5–2.5, and susceptible: mean score > 2.5.

### SSR markers and linkage analysis

SSR makers (Mdo.chr11.1–11.44) developed previously for the fine mapping of the *A* gene, responsible for the susceptibility of Japanese pear (*Pyrus pyrifolia* Nakai) to black spot disease [17], were used for initial marker enrichment and fine mapping the *Alt* locus (Additional file 2: Table S1). These markers were developed from contigs spanning 2.5–4.0 Mb of chr 11 of the apple genome version 1.0 primary assembly [44] corresponding to the *Alt* location. Moreover, novel SSR markers were developed from the same assembly. Batch Primer3 [45] was used to identify SSRs (Table 2) and for primer design. PCR amplification and detection of these markers were performed as described previously [46]. Linkage analysis and genetic map construction were performed using JoinMap version 4.1 [47]. Genetic distances between markers were calculated using a pseudo-testcross mapping strategy [48] by applying the regression mapping algorithm and the Kosambi’s map function. A minimum LOD score of 10.0 was used to establish the linkage groups.

### BAC library construction and shotgun sequencing

A BAC library of ‘SD’ was constructed as described previously [49]. Briefly, nuclear DNA isolated from ‘SD’ leaves was digested with *Hin*dIII restriction endonuclease. DNA fragments 100–180 kb in size at the first trial and slightly shorter than that at the second trial were ligated into the BAC vector pIndigoBAC-5 (Epicentre, Illumina), followed by transformation into *Escherichia coli*, ElectroMAX DH10B cells (Invitrogen, Life Technologies). Bacterial colonies were picked, transferred to LB medium dispensed to 384-well plates, and stored at − 80 °C.

To identify clones spanning the *Alt* region, four SSR markers (Mdo.chr11.3, Mdo.chr11.34, 3_34–12, and Mdo.chr11.33 m) were used to screen the BAC clones. PCR-amplified fragment length was used for the identification of *Alt*- or *alt*-associated clones. Because the *alt*-associated alleles of 3_34–12 and Mdo.chr11.33 m were not identified, these markers were able to detect only *Alt*-associated clones. DNA of selected BAC clones was randomly sheared and shotgun sequenced using the ABI 3730xl sequencer (Applied Biosystems, Life Technologies) with universal forward and reverse primers and the dye-terminator method. Shotgun sequences were assembled using PHRED and PHRAP software packages [50, 51]. To fill any gaps between assembled BAC contigs, additional shotgun sequencing was performed using other methods, such as dye-primer and transposon-tag sequencing [52].

### In silico gene prediction and characterization

Two BAC clones (SD-178_L15 and SD-241_O01) and partial sequence of a doubled haploid line of ‘Golden Delicious’ (GDDH13) [18], trimmed as to correspond to SD-178_L15, were subjected to in silico analysis. Nucleotide sequences of transposable elements identified using RepeatMasker (http://repeatmasker.org) were substituted as Ns prior to predicting open reading frames (ORFs). The ORF prediction was performed using FGENESH [53] with the algorithm for dicot plants. Predicted ORFs were queried against the nonredundant (nr) protein database of the National Center for Biotechnology Information (NCBI; https://blast.ncbi.nlm.nih.gov/) using BLASTP and searched for conserved domains using InterProScan [54]. Protein alignments were performed using the online versions of Needle and ClustalW available at the European Molecular Biology Laboratory (EMBL; http://www.ebi.ac.uk/Tools/psa/) and the DNA Data Bank of Japan (DDBJ; http://clustalw.ddbj.nig.ac.jp/index.php?lang=en), respectively.

### DNA marker for *Alt*

Three primers, “Alt-F” (5′-ATGTGTTTTATCCATCCAATTACG-3′), “Alt-R” (5′-AAGTTCAAATCTGACTCCGCTTA-3′), and “Alt-specific” (5′-GCCAGGGAGACTAAATTTTAAACTAAT-3′), were designed based on the sequence of the BAC clone SD-178_L15. PCRs were conducted with all three primers using the GoTaq Hot Start Master Mix (Promega, Madison, WI, USA). PCR conditions included an initial denaturation at 94 °C for 2 min, followed by 35 cycles of denaturation at 94 °C for 30 s, annealing at 55 °C for 30 s, and extension at 72 °C for 30 s, and a final extension at 72 °C for 10 min.

## Additional files

Additional file 1: **Figure S1.** Pedigree of susceptible apple accessions used in this study. (PDF 50 kb)

Additional file 2: **Table S1.** Mapping of SSR markers used in marker enrichment analysis. (PDF 30 kb)

Additional file 3: **Table S2.** Graphical illustration of genotypes showing recombination between *Alt* and flanking markers. (PDF 38 kb)

Additional file 4: **Figure S2.** Pairwise alignment of A6, a predicted ORF in the *Alt* region and XP_009379454.1, the gene most similar to A6, as revealed by a database search. (PDF 49 kb)

Additional file 5: **Figure S3.** Amino acid sequence alignment of predicted candidate genes of *Alt*, A8, a2, and G14. (PDF 37 kb)

Additional file 6: **Figure S4.** Amino acid sequence alignment of predicted candidate genes of *Alt*, A10, a4, and G16. (PDF 36 kb)

Additional file 7: **Table S3.** Presence of 12-bp insertion among founders and old cultivars. (PDF 50 kb)
