# Supplementary material for: Identification of candidate genes responsible for the susceptibility of apple (Malus × domestica Borkh.) to Alternaria blotch
Source: BMC Plant Biol. 2019 Apr 8;19:132. doi: 10.1186/s12870-019-1737-7 (PMC6454750; doi:10.1186/s12870-019-1737-7)
Supplement: Supplementary file 1 — Figure S1. Pedigree of susceptible apple accessions used in this study. (PDF 50 kb) [file 12870_2019_1737_MOESM1_ESM.pdf]

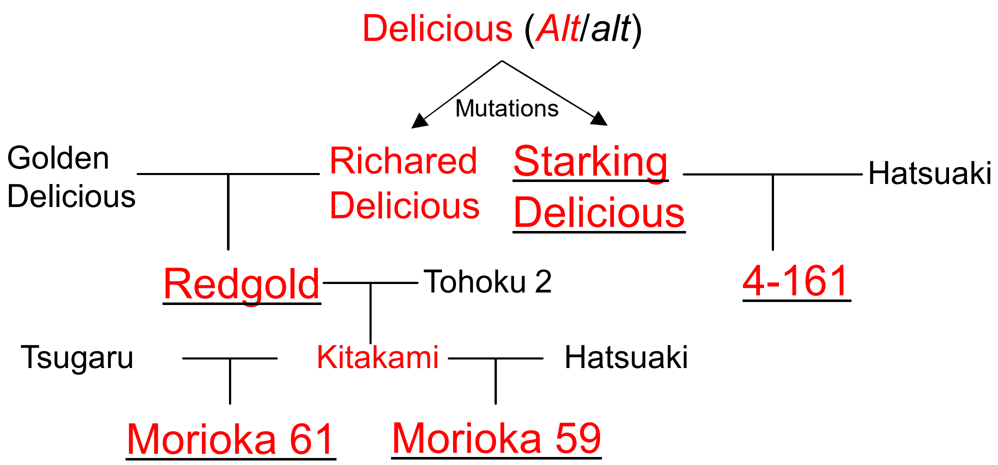

Fig. S1 Graphical representation of pedigree of apple accessions used for development of experimental populations.
Apples shown in red are susceptible to Alternaria blotch. Underlined apples are used as crossing parents for experimental populations.
