# Supplementary material for: Identification of candidate genes responsible for the susceptibility of apple (Malus × domestica Borkh.) to Alternaria blotch
Source: BMC Plant Biol. 2019 Apr 8;19:132. doi: 10.1186/s12870-019-1737-7 (PMC6454750; doi:10.1186/s12870-019-1737-7)
Supplement: Supplementary file 2 — Table S1. Mapping of SSR markers used in marker enrichment analysis. (PDF 30 kb) [file 12870_2019_1737_MOESM2_ESM.pdf]

Table S1 Mapping results of SSR markers used in marker enrichment analysis

| Markers | Amplification |
|---|---|
| Mdo.chr11.1 | Np |
| Mdo.chr11.2 | Np |
| Mdo.chr11.3 | Ok |
| Mdo.chr11.4 | Na |
| Mdo.chr11.5 | Ok |
| Mdo.chr11.6 | Ok |
| Mdo.chr11.7 | Na |
| Mdo.chr11.8 | Np |
| Mdo.chr11.9 | Ok |
| Mdo.chr11.10 | Ok |
| Mdo.chr11.11 | Ok |
| Mdo.chr11.12 | Ok |
| Mdo.chr11.13 | Np |
| Mdo.chr11.14 | Ok |
| Mdo.chr11.15 | Np |
| Mdo.chr11.16 | Ok |
| Mdo.chr11.17 | Np |
| Mdo.chr11.18 | Np |
| Mdo.chr11.19 | Np |
| Mdo.chr11.20 | Other |
| Mdo.chr11.21 | Other |
| Mdo.chr11.22 | Ok |
| Mdo.chr11.23 | Np |
| Mdo.chr11.24 | Np |
| Mdo.chr11.25 | Ok |
| Mdo.chr11.26 | Na |
| Mdo.chr11.27 | Ok |
| Mdo.chr11.28 | Ok |
| Mdo.chr11.29 | Other |
| Mdo.chr11.30 | Ok |
| Mdo.chr11.31 | Na |
| Mdo.chr11.32 | Na |
| Mdo.chr11.33 | Na |
| Mdo.chr11.34 | Ok |
| Mdo.chr11.35 | Ok |
| Mdo.chr11.36 | Na |
| Mdo.chr11.37 | Ok |
| Mdo.chr11.38 | Ok |
| Mdo.chr11.39 | Ok |
| Mdo.chr11.40 | Np |
| Mdo.chr11.41 | Ok |
| Mdo.chr11.42 | Np |
| Mdo.chr11.43 | Other |
| Mdo.chr11.44 | Ok |
| Mdo.chr11.45 | Np |

Ok: mapped on LG11
Np: not polymorphic
Na: not amplified
Other: mapped on other LG
