# Supplementary material for: Identification of candidate genes responsible for the susceptibility of apple (Malus × domestica Borkh.) to Alternaria blotch
Source: BMC Plant Biol. 2019 Apr 8;19:132. doi: 10.1186/s12870-019-1737-7 (PMC6454750; doi:10.1186/s12870-019-1737-7)
Supplement: Supplementary file 3 — Table S2. Graphical illustration of genotypes showing recombination between Alt and flanking markers. (PDF 38 kb) [file 12870_2019_1737_MOESM3_ESM.pdf]

Table S2 Graphical illustration of genotypes of individuals showing recombination between *Alt* and flanking markers

Markers/Trait		Mdo.chr11.27	Mdo.chr11.28	3_34-12 (288bp)	Mdo.chr11.3	Mdo.chr11.33m	3_34-12 (252bp)	Phenotype	3-34-18	Mdo.chr11.34	Mdo.chr11.35	Mdo.chr11.5	Mdo.chr11.38	Mdo.chr11.39	Mdo.chr11.41	Mdo.chr11.44
Origin of SSR in apple genome version 1.0 primary assembly		2903kb	2915kb	(3156kb)	3064kb	3157kb	3156kb		3160kb	3166kb	3201kb	3527kb	3564kb	3610kb	3670kb	3952kb
Alt (susceptibility) associated alleles (bp)		261	278	288	223	267	252		299	288	251	178	373	397	305	173
Number of recombinants with *Alt*		4	4	3	2	1	0		0	1	2	3	5	6	11	19
Plant ID	JSD_26							S								
	JSD_32							S								
	P13_7-3806		nn					S								
	P2_23							S								
	P7_7-2058		nn					S								
	P4_14							S								
	P3_2							S								
	P13_7-3743		nn					S								
	P13_7-3693		nn					R								
	P13_7-3709		nn					R								
	JSD_45							R								
	P4_5							R								
	P6_7-49		nn					R								
	P10_7-3141							R						nn		
	P1_38							R								
	P2_10							R								
	P4_37							R								
	P11_7-3284		nn					R								
	P1_35							R								
	P7_7-2120		nn					R								
	P6_7-50		nn					R								
	P13_7-3799		nn					R								
	P3_1							S								

nn indicates allele that was not identify whether the origin was susceptible or resistant parent.
