# Supplementary material for: Identification of candidate genes responsible for the susceptibility of apple (Malus × domestica Borkh.) to Alternaria blotch
Source: BMC Plant Biol. 2019 Apr 8;19:132. doi: 10.1186/s12870-019-1737-7 (PMC6454750; doi:10.1186/s12870-019-1737-7)
Supplement: Supplementary file 4 — Figure S2. Pairwise alignment of A6, a predicted ORF in the Alt region and XP_009379454.1, the gene most similar to A6, as revealed by a database search. (PDF 49 kb) [file 12870_2019_1737_MOESM4_ESM.pdf]

```
ORF_A6              1 MALGEVFLGAFLQLLLDRLTPREILEYFGNFRGVGQKLEKWRTTLSTIGA     50
                      ||||||||.||||||||||||||||||.||||||||||||||||||||||
XP_009379454.1      1 MALGEVFLAAFLQLLLDRLTPREILEYLGNFRGVGQKLEKWRTTLSTIGA     50

ORF_A6             51 VLSDAEERQLTEGGVKLWLHDLRDLAYDIEDMLDKFAIQMLKRMIEGRDQ    100
                      |||||||||||||||||||.|||||||||||||||||.||||||||||||
XP_009379454.1     51 VLSDAEERQLTEGGVKLWLDDLRDLAYDIEDMLDKFATQMLKRMIEGRDQ    100

ORF_A6            101 GSTSRK--------------------------------------------    106
                      .|.|||
XP_009379454.1    101 ASPSRKVRRSFYKVKLSFDMNSEMKKITKRLQDISERKDKFGLKDTGTSA    150

ORF_A6            107 --------------------------------------------------    106

XP_009379454.1    151 KESRSLPSSDVLDEKLVVGRDGDKGKIIELLSRKYEHADAVNFGVVAIVG    200

ORF_A6            107 --------------------------------------------------    106

XP_009379454.1    201 MAGVGKTTLAQLVFNNKDDAMKDIELKVWVSVSDDFDVVRVTKAILESIT    250

ORF_A6            107 -RP-----------------------------------------------    108
                       ||
XP_009379454.1    251 SRPVQVEEFSKMQHDLSEQLRGKKFLIVLDDIWDKDDYDLYDLWTRLQSP    300

ORF_A6            109 --------------------------------------------------    108

XP_009379454.1    301 FGVGAGGSKIIVTTRDRNVAKITGAAGVHNLECMADDDCMEIFERHAFGE    350

ORF_A6            109 --------------------------------------------------    108

XP_009379454.1    351 INNGKPVNYEVIRTKIVGKCCGLPLAARTLGGLLRRQEKEEWGEILDNKL    400

ORF_A6            109 --------------------------------------------------    108

XP_009379454.1    401 WNLADKSGLLPVLKLSYHYLPSHLKRCFAYCSILPNDYEFGEKQLVLLWM    450

ORF_A6            109 --------------------------------------------------    108

XP_009379454.1    451 AEGLIQQKHDDNKQMEDLGRDYFRELLARSLFQESSKNNSRYVMHDLVND    500

ORF_A6            109 --------------------------------------------------    108

XP_009379454.1    501 LARWAAGEICFRLEDKQGDNLQSTCFRRARHSSFIAGQFDGVMRFEDFPK    550

ORF_A6            109 --------------------------------------------------    108

XP_009379454.1    551 AERLRTSLPLSLSDSRGWYKYLSRKVTFELLPQLQYLRVLSFNGYRITEL    600
```

```
ORF_A6           109 -------------------------------------------------- 108

XP_009379454.1   601 PKSIGDLRLLQYLDLSYTLITSLPKSTSTLYYLQTLILEGCDELKSLPVN 650

ORF_A6           109 -------------------------------------------------- 108

XP_009379454.1   651 MSNLIKLRHLNNSGASSLEGMPLQLGRLTNLQSLPLFVASEGSDQSGIRE 700

ORF_A6           109 -------------------------------------------------- 108

XP_009379454.1   701 IGPLLHLRGALCLSRLENVTDVEDARRANLKCKERLDSLVLEWSRSRDTR 750

ORF_A6           109 -------------------------------------------------- 108

XP_009379454.1   751 ETESTVLDMLQPHTKIKELTIKRYAGKEFSSWVGGSFFSNMVLVRLEECN 800

ORF_A6           109 ---------------------------------GECILPFPLLEILEFLD 125
                                                      |||:|.|||||||||:|
XP_009379454.1   801 NCLLLPPLGQLPHLKELYIRGMNAVESVGPEFYGECVLAFPLLEILEFVD 850

ORF_A6           126 KQHWKVWLPFQLDHGSGCFPCLKRLSIKECSKLE---------------- 159
                     .||||||||||||||||.||||||||||:|||||
XP_009379454.1   851 MQHWKVWLPFQLDHGSGVFPCLKRLSIKKCSKLEGKLPENLDLLAKLEIV 900

ORF_A6           160 -----------------------------DAKVEFELLEYLCLSNISEVM 180
                                                  ||:|||||||.|.||||||||
XP_009379454.1   901 ECEELAVSIVNYKQLRQLNIKGCKVLVHTDAEVEFELLESLRLSNISEVM 950

ORF_A6           181 SLETGELFRKGLSKVTYLKISGCEKLMSSFKNEARLLQQLTSLGRLEIEG 230
                     ||:||||.|||||||..|||:|||||.||:|||.||||:||||||||||.
XP_009379454.1   951 SLQTGELLRKGLSKVRDLKINGCEKLTSSWKNEGRLLQRLTSLGRLEIED 1000

ORF_A6           231 NSRLVEELGEEAEELLQLQILECKLKFLELNKCENLVKLPKGLNQLSSLQ 280
                     |||||||||||||||||||||||||:||:|.|||||:|||||||||||||
XP_009379454.1  1001 NSRLVEELGEEAEELLQLQILECKLEFLQLKKCENLLKLPKGLNQLSSLQ 1050

ORF_A6           281 ELCIHECSSLVSFPDVGLPPSLKNIKIRKCDSLIYFAKFQIPQNLKIIDI 330
                     ||.|:||||||||||||||||||:|.||:|.||||||||||||||:.|:|
XP_009379454.1  1051 ELRIYECSSLVSFPDVGLPPSLKDIDIRRCHSLIYFAKFQIPQNLRRINI 1100

ORF_A6           331 GDCKSLKSLVDEEECERLGLIAPNGFFSDNTNHCLECIDMFGCQNLKSLP 380
                     .:|||||||||||||||:|||||||||:||||||||.|.::.||||||||
XP_009379454.1  1101 ENCKSLKSLVDEEECERVGLIAPNGFFNDNTNHCLEYIQIWNCQNLKSLP 1150

ORF_A6           381 DGLCHLSNLQELDIRECGSLVSILRLSGGRRPSNLIQIHIENCEKFEALP 430
                     ||||||||||.|.|.:|||||||.|||||||||||..|.|   .|..|||
XP_009379454.1  1151 DGLCHLSNLQTLIIGDCGSLVSIPRLSGGRRPSNLRWIVI---WKLVALP 1197
```

```
ORF_A6            431 EDMHNLNSLETLSIDYREGLIFPPNLTSLVIWKVKSY-------------     467
                      ||||||||||.|.|||||||.||||||||.||||||.
XP_009379454.1   1198 EDMHNLNSLEELRIDYREGLTFPPNLTSLTIWKVKSCKSLWELEWGLQRL    1247

ORF_A6            468 ---------PDTVSFPPNMVRMETLLPKSLTRLVINDFPNLKKLSSEGFQ     508
                               ||||||||:||||||||||||.|:|..|||||||||:||:
XP_009379454.1   1248 TSLIIYGEDPDTVSFPPDMVRMETLLPKSLTELIIVGFPNLKKLSSKGFE    1297

ORF_A6            509 FLTSLQYLGLWNCPKLASIPEEGLPPSLEELWIYGCPVLKERCQPGKGRY     558
                      ||||||.|.|..||||||||||.||||||||.||.|||||||||||||||
XP_009379454.1   1298 FLTSLQSLQLSKCPKLASIPEESLPPSLEELSIYRCPVLKERCQPGKGRY    1347

ORF_A6            559 WHKISHIPYIRIDDKEYSEDENEQKRYINGIAYFLSFHGHVFVWFFIKNG     608
                      ||||||||.|.||.:..
XP_009379454.1   1348 WHKISHIPCILIDCQPI---------------------------------    1364

ORF_A6            609 FAAFINHRQSFDNVVILSAPIEEHAGEVKFEAMVSSLPKEIVCDILLRLP     658

XP_009379454.1   1365 --------------------------------------------------    1364

ORF_A6            659 PKALIRCTSVSKPWNSMIKNPSVVHTHLRHTIDFNQFGTHHLLLRDNPVE     708

XP_009379454.1   1365 --------------------------------------------------    1364

ORF_A6            709 QNYYLHLDNHAFDEYCKLEYPSVSKRNRFPRVVYSLARGLWSSPRSFDPI     758

XP_009379454.1   1365 --------------------------------------------------    1364

ORF_A6            759 WCSLNDGRYQNFILAIDVDSDGNSIAMFTTTRGRNCPEDFLDIWVMKEYG     808

XP_009379454.1   1365 --------------------------------------------------    1364

ORF_A6            809 IEDSWAKLITLKSTGSGKKGALQALMF     835

XP_009379454.1   1365 ---------------------------    1364
```

Purple: RX-CC_like
Yellow: NB-ARC
Blue: Leucine-rich repeat domain superfamily
Red: F-box domain

Fig. S2 Pairwise alignment of deduced amino acid sequences between A6, a predicted ORF within *Alt* region, and XP_009379454.1.1, the gene most similar to A6, as revealed by a database search. Alignment was performed using Needle.
