# Supplementary material for: Identification of candidate genes responsible for the susceptibility of apple (Malus × domestica Borkh.) to Alternaria blotch
Source: BMC Plant Biol. 2019 Apr 8;19:132. doi: 10.1186/s12870-019-1737-7 (PMC6454750; doi:10.1186/s12870-019-1737-7)
Supplement: Supplementary file 5 — Figure S3. Amino acid sequence alignment of predicted candidate genes of Alt, A8, a2, and G14. (PDF 37 kb) [file 12870_2019_1737_MOESM5_ESM.pdf]

```
a2      MPLGEVFLAAFLQLLLDRLTPRDILNYFGNFRGVGKKLEKWRATLTTIAAVLSDADERQR 60
G14     MPLGEVFLAAFLQLLLDRLTPRDILNYFGNFRGVGKKLEKWRATLTTIAAVLSDADERQR 60
A8      MPLGEVFLAAFLQLLLDRLTPRDILNYFGNFRGVGKKLEKWRATLSTIAAVLSDADERQR 60
        *********************************************:**************

a2      TDDAVKLWLNNLRDLAYDVEDMLDKFATQMLKRTIEGRDQASTSNKVRNSLFKIKLNWNM 120
G14     TDDAVKLWLNNLRDLAYDVEDMLDKFATQMLKRTIEGRDQASTSNKVRNSLFKIKLNWNM 120
A8      TDDAVKLWLNNLRDLAYDVEDMLDKFATQMLKRTIEGRDQASTSNKVRNSLFKIKLNWDM 120
        **********************************************************:*

a2      NSEMKKITERLQDISDRKVKFSLKDTGTSAKESRSLPCSGVLDEKLLVGRDGDKGKIIEL 180
G14     NSEMKKITERLQDISDRKVKFSLKDTGTSAKESRSLPCSGVLDEKLLVGRDGDKGKIIEL 180
A8      NSEMKKITERLQDISDRKVKFGLKDTGTSAKESRSLPSSCVLDEKLVVGRDGDKGKIIEL 180
        *********************.***************.* ******:*************

a2      LKKSTNFGVVAIVGMPGVGKTTLAQLVFNNKDDAMKEFELKVWVSVSDDFDVVRVTKAIL 240
G14     LKKSTNFGVVAIVGMPGVGKTTLAQLVFNNKDDAMKEFELKVWVSVSDDFDVVRVTKAIL 240
A8      LKKSTNFGVVAIVGMPGVGKTTLAQLVFNNKDDAMKEFELKVWVSVSDDFDVVRVTKAIL 240
        ************************************************************

a2      ESMKPLPVQVEEFSKMQHDLSEQLRGKKFLIVLDDVWNKGDRDLNDLWLRLKSPFGVGAG 300
G14     ESMKPLPVQVEEFSKMQHDLSEQLRGKKFLIVLDDVWNKGDRDLNDLWLRLKSPFGVGAG 300
A8      ESMKPLPVQVEEFSKMQHDLSEQLRGKKFLIVLDDVWNKGDRDLNDLWLRLKSPFGVGAG 300
        ************************************************************

a2      GSKIIVTTRDLNVAKIMGATGFHNLECMANDDCLEIFERHAFGEVNSGKPVNYELIRTKI 360
G14     GSKIIVTTRDLNVAKIMGATGFHNLECMANDDCLEIFERHAFGEVNSGKPVNYELIRTKI 360
A8      GSKIIVTTRDLNVAKIMGATGFHNLECMANDDCLEIFERHAFGEVNSGKPVNYELIRTKI 360
        ************************************************************

a2      VEKCCGLPLAARTLGGLLRCKETDEWGEILDNKLWNLADNCNLPRVLELSYHYLPSHLKR 420
G14     VEKCCGLPLAARTLGGLLRCKETDEWGEILDNKLWNLADNCNLPRVLELSYHYLPSHLKR 420
A8      VEKCCGLPLAARTLGGLLRCKETDEWGEILDNKLWNLADNCNLPRVLELSYHYLPSHLKR 420
        ************************************************************

a2      CFAYCSILPNDYEFGEKQLVLLWMAEGLIQQKPDNNKQMEDLGRDYFRELLARSLFQESS 480
G14     CFAYCSILPNDYEFGEKQLVLLWMAEGLIQQKPDNNKQMEDLGRDYFRELLARSLFQESS 480
A8      CFAYCSILPNDYEFGQKQLVLLWMAEGLIQQKPDNNKQMEDLGRDYFRELLARSLFQESS 480
        ***************:********************************************

a2      KNNSRYVMHDLVSELTRWAAGEICFRLEDKQGDNLQSTCFRGARHSSFIAGEFDGVKRFE 540
G14     KNNSRYVMHDLVSELTRWAAGEICFRLEDKQGDNLQSTCFRGARHSSFIAGEFDGVKRFE 540
A8      KNNSRYVMHDLVSELTRWAAGEICFRLEDKQGDNLQSTCFRGARHSSFIAGEFDGVKRFE 540
        ************************************************************
```

```
a2      DFPKAERLRTFLPLSLSYSTGWLKFLSRQVTFKLLPQLQYLRVLSFNDYKITALPESIGD 600
G14     DFPKAERLRTFLPLSLSYSTGWLKFLSRQVTFKLLPQLQYLRVLSFNDYKITALPESIGD 600
A8      DFPKAERLRTFLPLSLSYSTGWLKFLSRQVTFKLLPQLQYLRVLSFNDYKITALPESIGD 600
        ************************************************************

a2      LRLLQYLDLSYTYITSLPKSISTLYNLQTLILEHCYYLKSLLADMSNLINLRHLNNSYVS 660
G14     LRLLQYLDLSYTYITSLPKSISTLYNLQTLILEHCYYLKSLLADMSNLINLRHLNNSYVS 660
A8      LRLLQYLDLSYTYITSLPKSISTLYNLQTLILEHCYYLKSLLADMSNLINLRHLNNSYVS 660
        ************************************************************

a2      SLEGMPPNLGSLVNLQSLPNFVVSGGSDQSGIREIGPLSHLRGTLCISGLENVTDVEDAR 720
G14     SLEGMPPNLGSLVNLQSLPNFVVSGGSDQSGIREIGPLSHLRGTLCISGLENVTDVEDAR 720
A8      SLEGMPPNLGSLVNLQSLPNFVVSGGSDQSGIREIGPLSHLRGTLCISGLENVTDVEDAR 720
        ************************************************************

a2      RANLKCKERLDSLVLKWSHSSDTRETESVVLDMLEPHTKLKELTIKSYAGEEFSSWVGGP 780
G14     RANLKCKERLDSLVLKWSHSSDTRETESVVLDMLEPHTKLKELTIKSYAGEEFSSWVGGP 780
A8      RANLKCKERLDSLVLKWSHSSDTRETESVVLDMLEPHTKLKELTIKSYAGEEFSSWVGGP 780
        ************************************************************

a2      LFSNMVLVCLQKCNNCLSLPPLGQLPHLRELYISGMNAVESVGAEFYGERMLPFPVLETL 840
G14     LFSNMVLVCLQKCNNCLSLPPLGQLPHLRELYISGMNAVESVGAEFYGERMLPFPVLETL 840
A8      LFSNMVLVCLQKCNNCLSLPPLGQLPHLRELYISGMNAVESVGAEFYGERMLPFPVLETL 840
        ************************************************************

a2      EFVEMQHWKEWLPFQADHEGGAFPCLKTLLVKKCSKLEGKLPENLNSLAKLKIVECEELV 900
G14     EFVEMQHWKEWLPFQADHEGGAFPCLKTLLVKKCSKLEGKLPENLNSLAKLKIVECEELV 900
A8      EFVEMQHWKEWLPFQADHEGGAFPCLKTLLVKKCSKLEGKLPENLNSLAKLKIVECEELV 900
        ************************************************************

a2      VSIANCKQLRQLNIDGCKVLVHTAAKVEFELLESLCLSNILEMMSLQTGELLKKGLTKVR 960
G14     VSIANCKQLRQLNIDGCKVLVHTAAKVEFELLESLCLSNISEMMSLQTGELLKKGLTKVR 960
A8      VSIANCKQLRQLNIDGCKVLVHTAAKVEFELLESLCLSNISEMMSLQTGELLKKGLTKVR 960
        **************************************** *******************

a2      DLKISGCEELTSSLKNEKGRLLQQLTSLGSLEIEDNSRLVEELGKEAEELQISKCKLEFL 1020
G14     DLKISGCEEVTSSLKNEEGRLLQQLTSLGSLEIEDNSRLVEELGKEAEELQISECKLEFL 1020
A8      DLKISGCEEVTSSLKNEEGRLLQQLTSLGSLEIEDNSRLVEELGKEAEELQISECKLEFL 1020
        *********:*******:***********************************:******

a2      ELKKCENLSKLLKGLNQLSSLQELRIHECSSLVSFPDVGLPSLKDIQITSCDSLIYFAKF 1080
G14     ELKKCENLSKLLKGLNQLSSLQELRIHECSSLVSFPDVGLPSLKDIQITSCDSLIYFAKF 1080
A8      ELKKCENLSKLLKGLNQLSSLQELRIHECSSLVSFPDVGLPSLKDIQITSCDSLIYFAKF 1080
        ************************************************************
```

```
a2              QIPQNLRRTEIKDCKSLKSLVDEEAVSSSWSSFSHSCLEYLRIRGCESLTSLSLSGQLPR 1140
G14             QIPQNLRRTEIKDCKSLKSLVDEEAVGSSWSSFSHSCLEYLRIRGCESLMSLSLSGQLPR 1140
A8              QIPQNLRRTEIKDCKSLKSLVDEEAVGSSWSSFSHSCLEYLRIRGCESLMSLSLSGQLPR 1140
                **************************.********************** **********

a2              TLKHLDLENCDRLELIAGDGFFRDNTNDCLEYIRIWNCQNLKSLPDGLCHLSNLQTLGIG 1200
G14             TLKHLDLENCDRLELIAGDGFFRDNTNDCLEYIRIWNCQNLKSLPDGLCHLSNLQTLGIG 1200
A8              TLKHLDLENCDRLELIAGDGFFRDNTNDCLEYIRIWNCQNLKSLPDGLCHLSNLQTLGIG 1200
                ************************************************************

a2              NCGSLVSIPRLSGGRRPSNLRKIWIIGCNKLEALPEDMHNLNSLEELSIDYREGLTFPPN 1260
G14             NCGSLVSIPRLSGGRRPSNLRKIWIIGCNKLEALPEDMHNLNSLEELSIDYREGLTFPPN 1260
A8              NCGSLVSIPRLSGGRRPSNLRKIWIIGCNKLEALPEDMHNLNSLEELSIDYREGLTFPPN 1260
                ************************************************************

a2              LTSLEIGKVKSCKSLWELEWGLHRLTSLRELGIRSTDPDTVSFPPDMVRMETLFPKSLTN 1320
G14             LTSLEIGKVKSCKSLWELEWGLHRLTSLRELGIRSTDPDTVSFPPDMVRMETLFPKSLTN 1320
A8              LTSLEIGKVKSCKSLWELEWGLHRLTSLRELGIRSTDPDTVSFPPDMVRMETLFPKSLTN 1320
                ************************************************************

a2              LSIDGFPNLKKLSSKGFQFLTSLQSLDLSNCPKLASIPEEGLPPSLERLTIYECPVLKER 1380
G14             LSIDGFPNLKKLSSKGFQFLTSLQSLDLSNCPKLASIPEEGLPPSLERLTIYECPVLKER 1380
A8              LSIDGFPNLKKLSSKGFQFLTSLQSLDLSNCPKLASIPEEGLPPSLERLTIYECPVLKER 1380
                ************************************************************

a2              CQPGKGRYWHKISHIPFIWIDYKQM 1405
G14             CQPGKGRYWHKISHIPFIWIDYKQM 1405
A8              CQPGKGRYWHKISHIPFIWIDYKQM 1405
                *************************
```

Purple: RX-CC_like

Yellow: NB-ARC

Blue: Leucine-rich repeat domain superfamily

Fig. S3 Amino acid sequence alignment of predicted ORFs of A8, a2 and G14. Alignment was performed using ClastalW 2.1.
