# Supplementary material for: Identification of candidate genes responsible for the susceptibility of apple (Malus × domestica Borkh.) to Alternaria blotch
Source: BMC Plant Biol. 2019 Apr 8;19:132. doi: 10.1186/s12870-019-1737-7 (PMC6454750; doi:10.1186/s12870-019-1737-7)
Supplement: Supplementary file 6 — Figure S4. Amino acid sequence alignment of predicted candidate genes of Alt, A10, a4, and G16. (PDF 36 kb) [file 12870_2019_1737_MOESM6_ESM.pdf]

```
A10             MSQKLTTNRKFEKGRPLPKFGEWDVNDPASAEGFTVIFNKARDEKKTGGKPESPGKVDPN 60
G16             MSQKLTTNRKFEKGRPLPKFGEWDVNDPASAEGFTVIFNKARDEKKTGGKPESPGKVDPN 60
a4              MSQKLTTNRKFEKGRPLPKFGEWDVNDPASAEGFTVIFNKARDEKKTGGKPESPGKVDPN 60
                ************************************************************

A10             IRHGGGVDPGTGRPSSESFRFTCKSQFNAYAQKHKHGARIEVLDSGHPKLPTEKAAKWEN 120
G16             IRHGGGVDPGTGRPSSESFRFTCKSQFNAYAQKHKHGARIEVLDSGHPKLPTEKAAKWEN 120
a4              IRHGGGVDPGTGRPSS-------------------------------VRLWSPQASVKE- 88
                ****************                                :* : :*:  *

A10             LGSTGTDKRCRYEKRPYGSTSDPLLRLLHQRLQETIYITKRLRKKLCLRGAREL 174
G16             LGSTGTDKRCRYEKRPYGSTSDPLLRLLHQRLQETIYITKRLRKKLCLRGAREL 174
a4              -----------------GSEVGKLGELWHR--QALLTRKKALWIDL-------- 115
                                 **  . * .* *:  *  :  .* *  .*
```

Red: RIN4, pathogenic type III effector avirulence factor Avr cleavage site

Fig. S4 Amino acid sequence alignment of predicted ORFs of A10, a4 and G16. Alignment was performed using ClastalW 2.1.
