# Supplementary material for: Identification of candidate genes responsible for the susceptibility of apple (Malus × domestica Borkh.) to Alternaria blotch
Source: BMC Plant Biol. 2019 Apr 8;19:132. doi: 10.1186/s12870-019-1737-7 (PMC6454750; doi:10.1186/s12870-019-1737-7)
Supplement: Supplementary file 7 — Table S3. Presence of 12-bp insertion among founders and old cultivars. (PDF 50 kb) [file 12870_2019_1737_MOESM7_ESM.pdf]

Table S3 Presence of the 12-bp insertion among founders and old cultivars

Cultivars	Sympton scores of *A . alternata* apple pathotype inoculation test[a]	Presence of the 12-bp insertion	Origin	
Starking Delicious	3.9	+	USA, 1800s	Founder
Jonathan	0	-	USA, 1800s	Founder
Golden Delicious	0.8	-	USA, 1800s	Founder
Ralls Janet	1.2	-	USA, 1800s	Founder
Worcester Pearmain	0	-	USA, 1800s	Founder
Indo	4.1	+	Japan, 1800s	Founder
Cox's Orange Pippin	Resistant[b]	-	UK, 1800s	Founder
McIntosh	0.2	-	Canada, 1800s	Founder
Alexander	0.0	-	Uklaine, 1700s	
American Summer Pearmain	0.0	-	USA, 1800s	
Baldwin	3.2	+	USA, 1700s	
Ben Davis	0.0	-	USA, 1800s	
Benfle	0.0	-	Unknown, ?	
Blue Pearmain	0.2	-	USA, 1800s	
Bramley's Seedling	3.2	+	UK, 1800s	
Calville Rouge	3.0	+	France?, ?	
Carolina Red June	0.0	-	USA, 1800s	
Cogswell	1.4	-	USA, 1700s	
Duchess of Oldenburg	0.0	-	Russia, 1700s	
Early Harvest	0.2	-	USA, 1800s	
Early Joe	4.2	+	USA, 1800s	
Early Strawberry	4.2	+	USA, 1800s	
Esopus Spitzenburgh	3.8	+	USA, 1700s	
Fameuse	0.0	-	Canada, 1700s	
Gravenstein	0.0	-	Denmark, 1600s	
Jersey Sweet	0.2	-	USA, 1800s	
King of Tompkins	3.2	+	USA, 1800s	
Northern Spy	4.0	+	USA, 1800s	
Orange Pippin	0.2	-	UK, 1700-1800s	
Porter	3.4	+	USA, 1800s	
Prinzen Apfel	0.0	-	Germany, 1700s	
Rome Beauty	0.0	-	USA, 1800s	
Roxbury Russet	0.2	-	USA, 1600s	
Sops of Wine	0.2	-	UK, 1800s	
Summer Queen	3.0	+	USA, 1800s	
Swaar	3.8	+	USA, 1800s	
Tallman's Sweet	0.2	-	USA, 1800s	
Twenty Ounce	0.2	-	USA, 1800s	
Wagener	2.2	-	USA, 1700s	
Wealthy	0.0	-	USA, 1800s	
White Winter Pearmain	4.2	+	USA, 1800s	
Winesap	0.4	-	USA, 1800s	
York Imperial	0.8	-	USA, 1800s	

[a]Adopted from Abe et al. [15].

[b]No visible sympton by Tsuchiya et al. [34]
